# Supplementary material for: An all-photonic full color RGB system based on molecular photoswitches
Source: Nat Commun. 2019 Sep 5;10:3996. doi: 10.1038/s41467-019-11885-4 (PMC6728369; doi:10.1038/s41467-019-11885-4)
Supplement: Supplementary file 1 — Supplementary Information [file 41467_2019_11885_MOESM1_ESM.pdf]

SUPPLEMENTARY INFORMATION:

An all-photonic full color RGB system based on molecular photoswitches

Naren et al.

Supplementary Note 1. Synthesis

Experimental procedures for the preparation of DAEg, DAEr, and the ST-7-8 copolymer have been reported previously.[1, 2] Perylene (per) was purchased from Sigma-Aldrich and used without further purification.

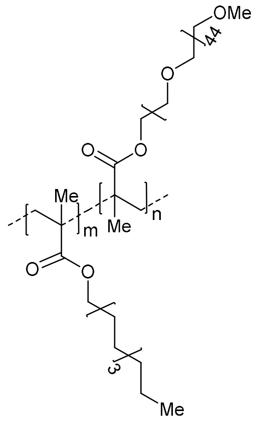

ST-7-8

Supplementary Figure 1. Structure of the ST-7-8 copolymer. m:n = 4:1

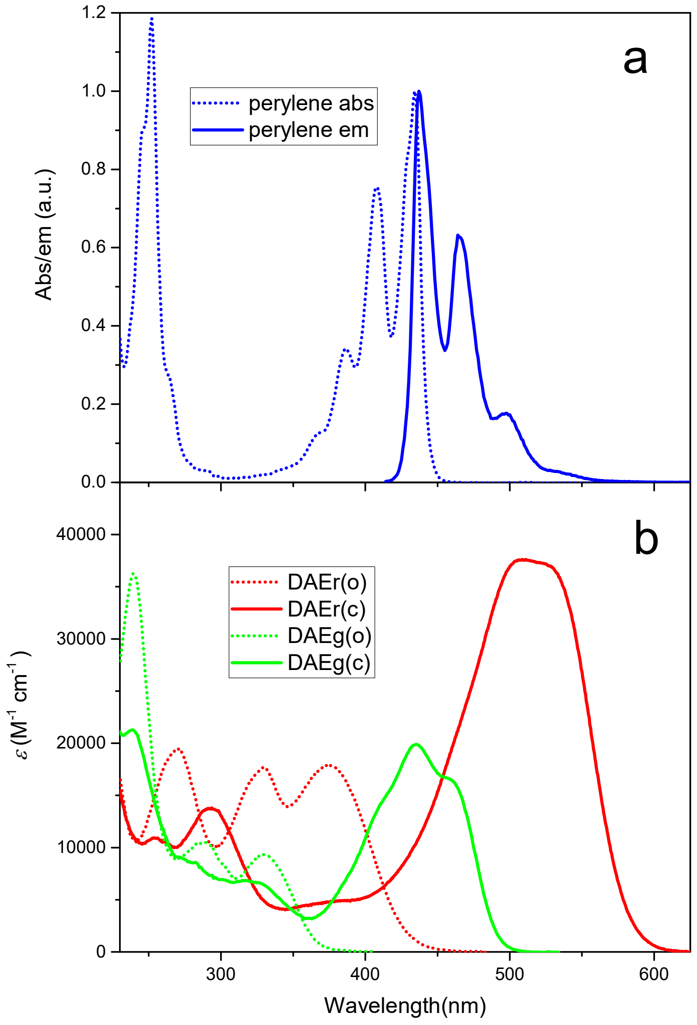

Supplementary Figure 2. Spectral properties. Absorption spectra (in acetonitrile) of the DAE derivatives in the open and closed isomeric forms (b) together with the absorption and emission spectra of per (a), also in acetonitrile.

Supplementary Note 2. Steady-state emission and time resolved single photon counting (SPC) data of the micelle cocktails

Supplementary Figures 3-9 below are organized as follows:

Left panel: Steady-state emission (λ_{exc} = 423 nm). Per alone (black line), and the per + DAE cocktails (red line). 423 nm was selected as to differentiate (red shifting) the excitation wavelength for emission readout as much as possible from the 242 nm and 415 nm light, at the same time avoiding influence from scattering in the emission spectra.

Right panel: Time-resolved (SPC) fluorescence decays of per (λ_{exc} = 405 nm, λ_{em} = 440 nm). Per alone (black line) and the per + DAE cocktails (red line).

The following parameters are also shown for each set of measurements:

Quenching efficiency of per in the cocktail from steady-state measurements ($E_{quench, SS}$)

Quenching efficiency of per in the cocktail from SPC measurements ($E_{quench, SPC}$)

Per + DAEg(o) + DAEr(o)

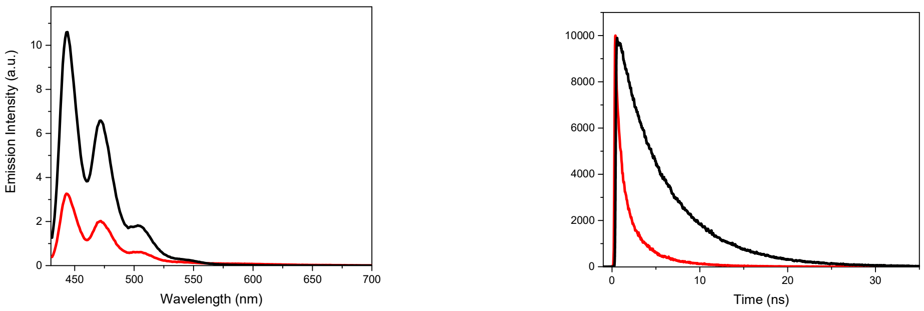

Supplementary Figure 3. [per]= 0.45 µM, [DAEg]= 3.7 µM, and [DAEr]= 1.1 µM.

Decay	A_1	τ_1(ns)	A_2	τ_2(ns)	A_3	τ_3(ns)	τ_{avg}(ns)	χ^2
Per alone (black)	9603	5.6	1895	0.80	_	_	4.8	1.16
Per in cocktail (red)	5057	1.2	8705	0.24	1710	3.7	0.96	1.0

($E_{quench, SS}$) = 0.69
($E_{quench, SPC}$) = 0.80

Per + DAEg(c) + DAEr(o)

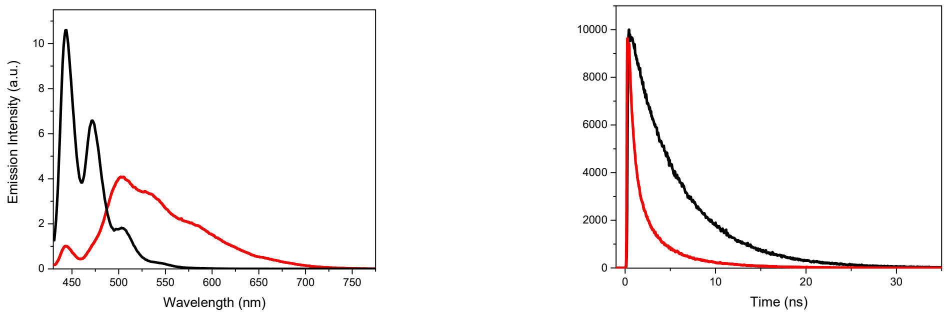

Supplementary Figure 4. [per]= 0.45 µM, [DAEg]= 3.7 µM, and [DAEr]= 1.1 µM.

Decay	A_1	τ_1(ns)	A_2	τ_2(ns)	A_3	τ_3(ns)	τ_{avg}(ns)	χ^2
Per alone (black)	9603	5.6	1895	0.80	_	_	4.8	1.16
Per in cocktail (red)	2029	4.5	5530	1.2	12799	0.20	0.90	1.09

($E_{quench, SS}$) = 0.90
($E_{quench, SPC}$) = 0.81

Per + DAEg(o) + DAEr(c)

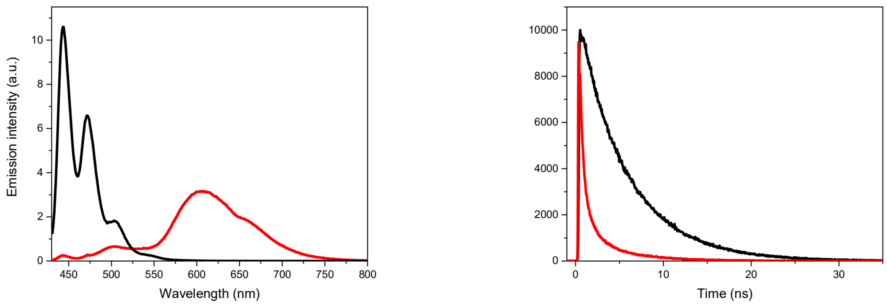

Supplementary Figure 5. [per]= 0.45 µM, [DAEg]= 3.7 µM, and [DAEr]= 1.1 µM.

Decay	A_1	τ_1(ns)	A_2	τ_2(ns)	A_3	τ_3(ns)	τ_{avg}(ns)	χ^2
Per alone (black)	9603	5.6	1895	0.80	_	_	4.8	1.16
Per in cocktail (red)	1243	4.6	4439	0.95	18784	0.14	0.51	1.05

($E_{quench, SS}$) = 0.98
($E_{quench, SPC}$) = 0.89

Per + DAEg(o)

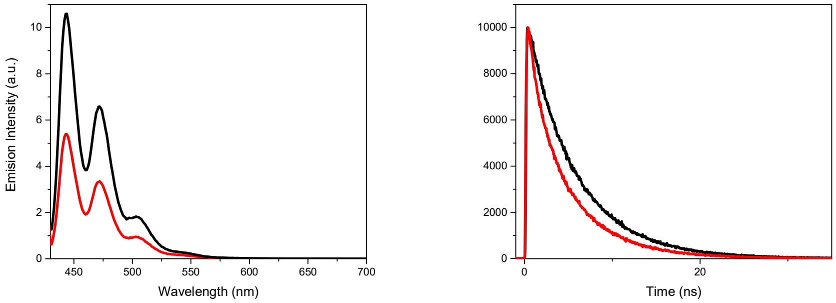

Supplementary Figure 6. [per]= 0.45 μM, [DAEg]= 3.7 μM.

Decay	A_1	τ_1(ns)	A_2	τ_2(ns)	A_3	τ_3(ns)	τ_{avg}(ns)	χ^2
Per alone (black)	9603	5.6	1895	0.80	_	_	4.8	1.16
Per in cocktail (red)	7898	5.1	4456	1.0	_	_	3.6	1.39

($E_{quench,\ SS}$) = 0.49
($E_{quench,\ SPC}$) = 0.24

Per + DAEg(c)

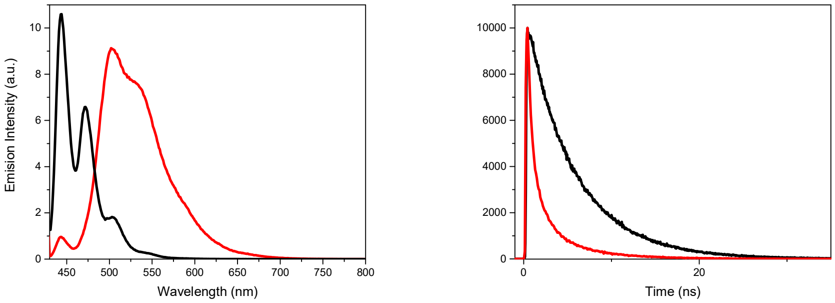

Supplementary Figure 7. [per]= 0.45 μM, [DAEg]= 3.7 μM.

Decay	A_1	τ_1(ns)	A_2	τ_2(ns)	A_3	τ_3(ns)	τ_{avg}(ns)	χ^2
Per alone (black)	9603	5.6	1895	0.80	_	_	4.8	1.16
Per in cocktail (red)	2699	4.8	5466	1.4	14964	0.26	1.1	1.0

($E_{quench,\ SS}$) = 0.91
($E_{quench,\ SPC}$) = 0.78

Per + DAEr(o)

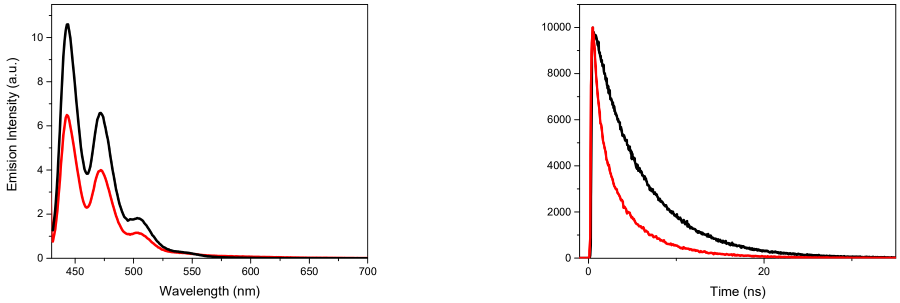

Supplementary Figure 8. [per]= 0.45 µM, [DAEr]= 1.1 µM.

Decay	A_1	τ_1(ns)	A_2	τ_2(ns)	A_3	τ_3(ns)	τ_{avg}(ns)	χ^2
Per alone (black)	9603	5.6	1895	0.80	_	_	4.8	1.16
Per in cocktail (red)	5062	4.4	9033	0.76	_	_	2.1	1.93

$(E_{quench,\ SS}) = 0.39$
$(E_{quench,\ SPC}) = 0.57$

Per + DAEr(c)

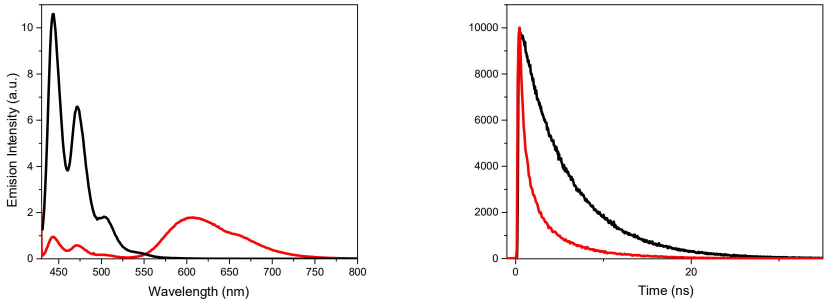

Supplementary Figure 9. [per]= 0.45 µM, [DAEr]= 1.1 µM.

Decay	A_1	τ_1(ns)	A_2	τ_2(ns)	A_3	τ_3(ns)	τ_{avg}(ns)	χ^2
Per alone (black)	9603	5.6	1895	0.80	_	_	4.8	1.16
Per in cocktail (red)	2136	4.9	5241	1.3	19167	0.18	0.78	1.0

$(E_{quench,\ SS}) = 0.91$
$(E_{quench,\ SPC}) = 0.84$

Supplementary Note 3. Redox data and driving forces for photoinduced electron transfer (PET) reactions

Listed below are the following parameters:

E_{00} = energy of the lowest excited singlet state

E_{ox} = first oxidation potential value (vs SCE)

E_{red} = first reduction potential value (vs SCE)

Per:[3] E_{00} = 2.81 eV (441 nm), E_{ox} = 1.03 V, E_{red} = -1.72 V

DAEg(o): E_{00} = 3.76 eV (330 nm), E_{ox} = 2.43 V, E_{red} = -0.97 V

DAEg(c): E_{00} =2.56 eV (484 nm), E_{ox} = 1.26 V, E_{red} = -0.47 V

DAEr(o): E_{00} = 2.84 eV (436nm), E_{ox} = 1.57 V, E_{red} = -1.27 V

DAEr(c): E_{00} =2.15 eV (576 nm), E_{ox} = 1.20 V, E_{red} = -0.55 V

The corresponding free energy changes for PET were determined using the following equation:

$$\Delta G^0 = e(E_{ox} - E_{red}) - E_{00} \quad (1)$$

Reaction	ΔG^0 (eV)
Per* + DAEr(o) → Per$^-$ + DAEr(o)$^+$	+0.48
Per* + DAEr(o) → Per$^+$ + DAEr(o)$^-$	-0.51
Per + DAEr(o)* → Per$^+$ + DAEr(o)$^-$	-0.54
Per + DAEr(o)* → Per$^-$ + DAEr(o)$^+$	+0.45
Per* + DAEr(c) → Per$^-$ + DAEr(c)$^+$	+0.11
Per* + DAEr(c) → Per$^+$ + DAEr(c)$^-$	-1.23
Per + DAEr(c)* → Per$^+$ + DAEr(c)$^-$	-0.57
Per + DAEr(c)* → Per$^-$ + DAEr(c)$^+$	+0.79
Per* + DAEg(o) → Per$^-$ + DAEg(o)$^+$	+1.30
Per* + DAEg(o) → Per$^+$ + DAEg(o)$^-$	-0.85
Per + DAEg(o)* → Per$^+$ + DAEg(o)$^-$	-1.76
Per + DAEg(o)* → Per$^-$ + DAEg(o)$^+$	+0.39
Per* + DAEg(c) → Per$^-$ + DAEg(c)$^+$	+0.13
Per* + DAEg(c) → Per$^+$ + DAEg(c)$^-$	-1.35
Per + DAEg(c)* → Per$^+$ + DAEg(c)$^-$	-1.06
Per + DAEg(c)* → Per$^-$ + DAEg(c)$^+$	+0.42
DAEg(c)* + DAEr(c) → DAEg(c)$^+$ + DAEr(c)$^-$	-0.75
DAEg(c)* + DAEr(c) → DAEg(c)$^-$ + DAEr(c)$^+$	-0.89
DAEg(c) + DAEr(c)* → DAEg(c)$^-$ + DAEr(c)$^+$	-0.48
DAEg(c) + DAEr(c)* → DAEg(c)$^+$ + DAEr(c)$^-$	-0.34

Supplementary Note 4. Estimation of the donor-acceptor distances in the micelles

The calculations are based on the assumption that any excited state intermolecular reaction (e.g. PET or FRET) most likely will occur with the closet acceptors. The average nearest donor-acceptor distance R_{mp} is calculated by using the equations below:

$$a = (\frac{3}{4\pi n})^{1/3} \quad (2)$$

$$R_{mp} = (2/3)^{1/3}a \approx 0.8735a \quad (3)$$

Here, a is the Wigner-Seitz radius and n is the number of molecules per cubic meter (here taken as the sum of the donor and the acceptor concentrations). The estimated effective hydrophobic core size of the micelles (15 nm diameter, estimated from the hydrodynamic diameter given below) is used in the determination of the micelle volume. The bulk concentration of the individual compounds and the concentration of the micelles results in the following estimated average donor-acceptor distances:

$R_{D\text{-}A}$(per-DAEg) = 28 Å
$R_{D\text{-}A}$(per-DAEr) = 38 Å

This in turn implies expected FRET efficiencies of 95% and 84%, respectively. This is to be compared to the experimentally obtained values of around 90% for both situations.

The following data was used in the calculations:

Bulk concentrations: [per] = 0.45μM, [DAEg] = 3.7μM, [DAEr] = 1.1 μM, and [micelles] = 0.31 μM)

The average molecular weight of the individual micelles[4] = 580 kDa
The average hydrodynamic diameter of the micelles[4] = 26 nm

Finally, at the mM concentrations of the fluorophores inside the micelles, per is expected to display the red-shifted excimer emission. This is clearly not observed here, which strongly supports our calculation that every micelle contains on the average no more than 1 per unit.

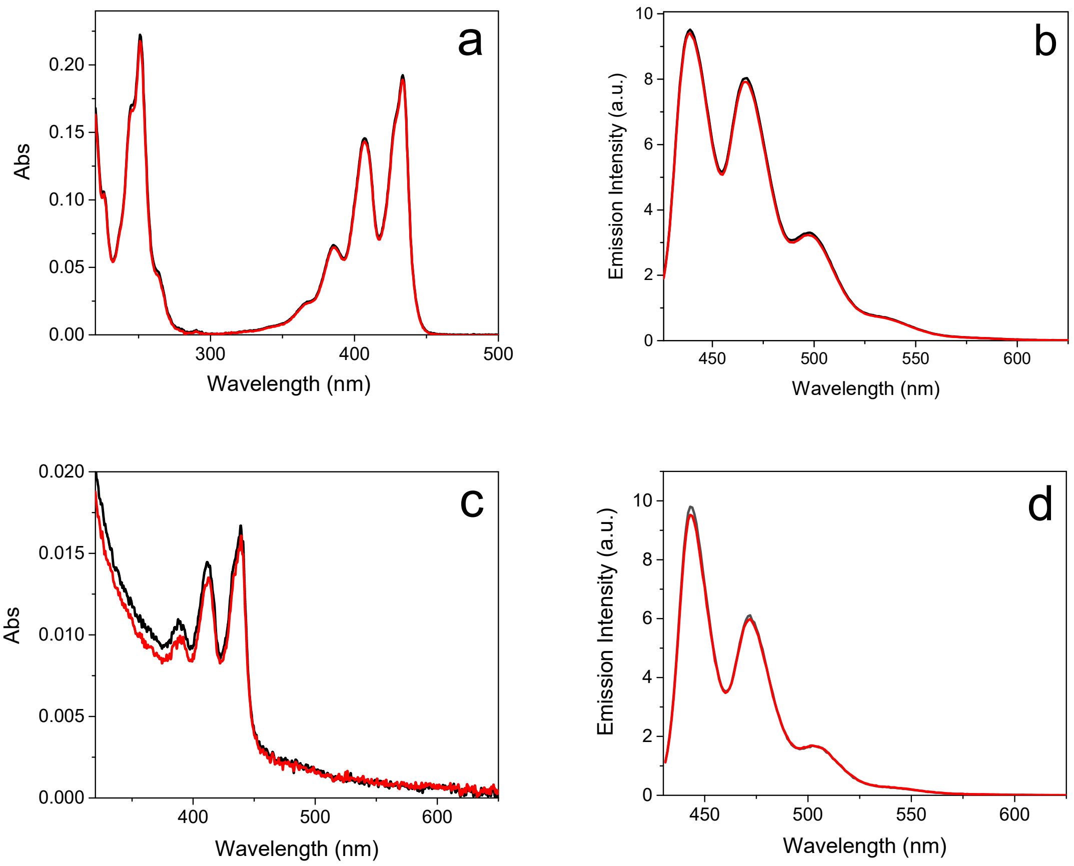

Supplementary Figure 10. Photostability of per. Absorption (a and c) and emission (b and d) of per before (black lines) and after (red lines) 160 min irradiation at 405 nm (1 mWcm^{-2}). a and b in acetonitrile, c and d in the polymer micelles. Please note the pronounced scattering from the micelles in c.

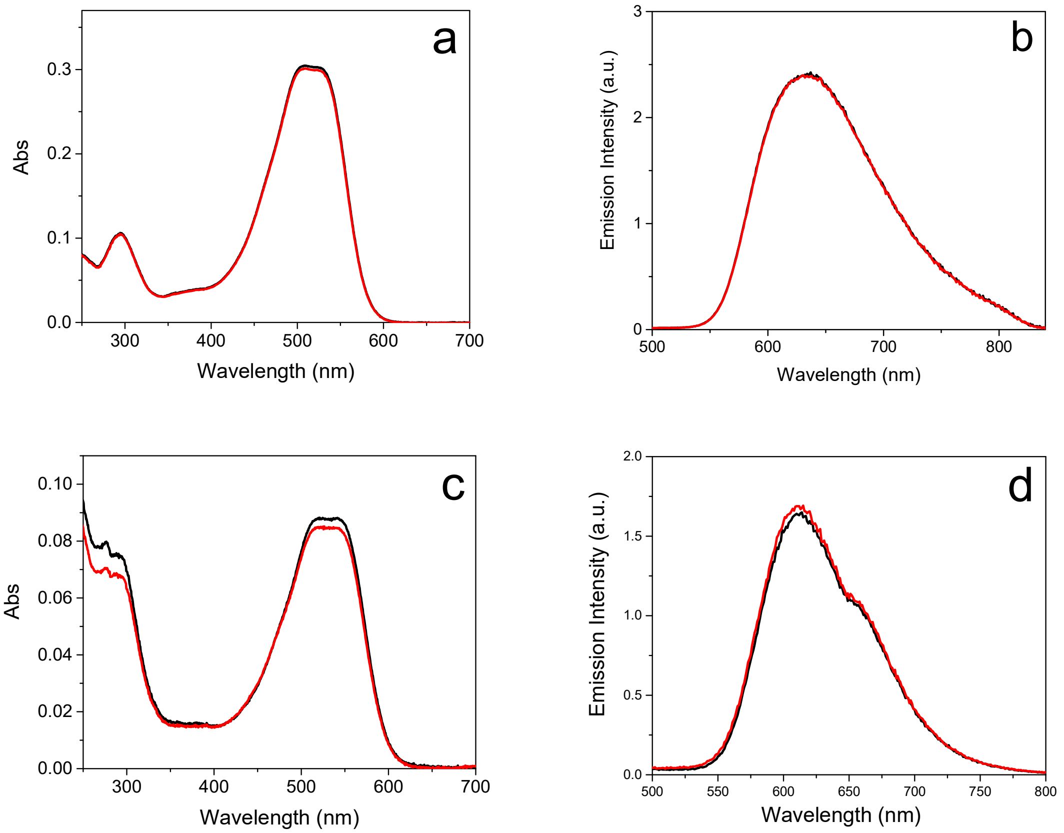

Supplementary Figure 11. Photostability of DAEr. Absorption (a and c) and emission (b and d) of DAEr(c) before (black lines) and after (red lines) 160 min irradiation at 365 nm (0.7 $mWcm^{-2}$). a and b in acetonitrile, c and d in the polymer micelles. Please note the pronounced scattering from the micelles in c.

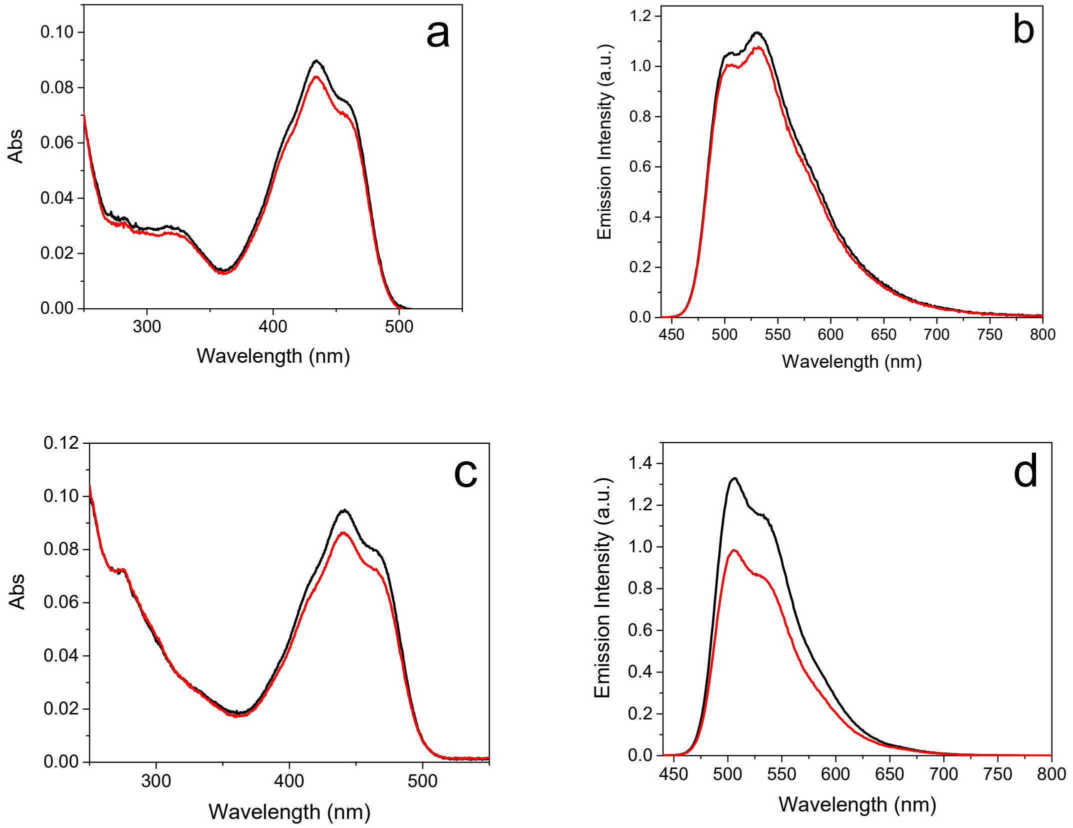

Supplementary Figure 12. Photostability of DAEg. Absorption (a and c) and emission (b and d) of DAEg(c) before (black lines) and after (red lines) irradiation at 365 nm (0.7 $mWcm^{-2}$). a and b in acetonitrile (285 min. irradiation), c and d in the polymer micelles (160 min. irradiation). Please note the pronounced scattering from the micelles in c. It is seen that extensive light exposure results in significantly decreased absorption and emission intensity, varying between a 6% and a 25% decrease (absorption in acetonitrile and emission in the polymer micelles, respectively).

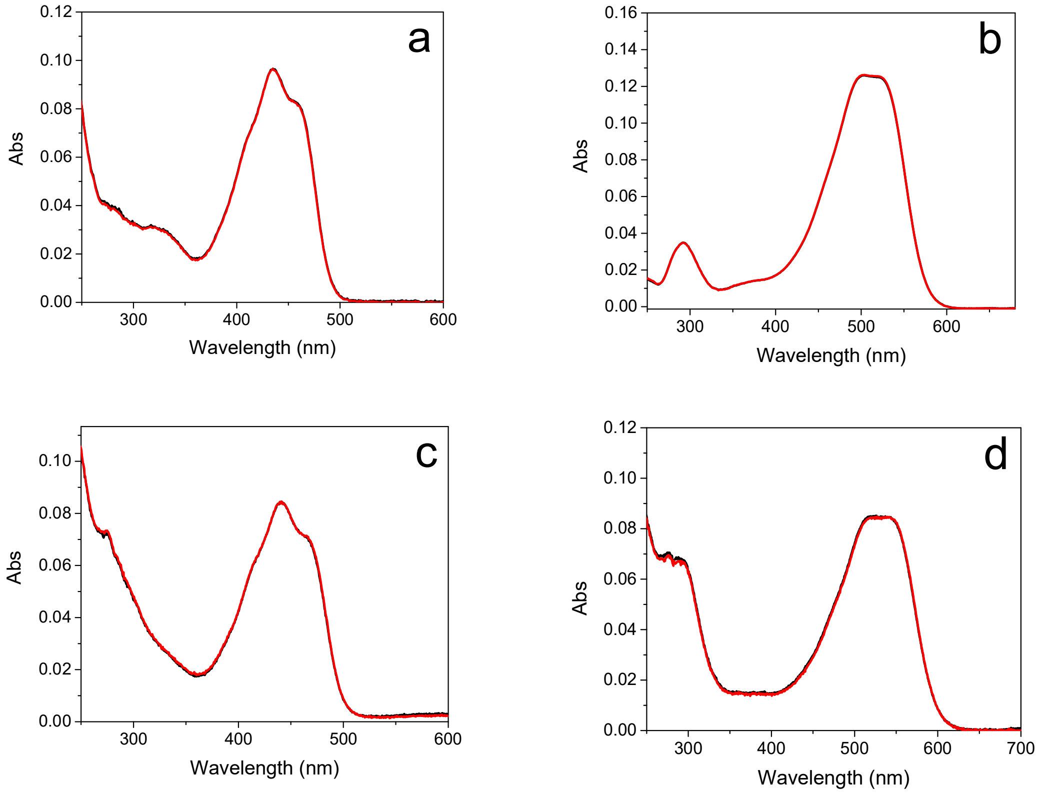

Supplementary Figure 13. Thermal stability. Absorption spectra of the closed forms of the DAE photoswitches before (black lines) and after (red lines) 20 h. in the dark. a and b in acetonitrile, c and d in the polymer micelles. a and c: DAEg(c), b and d: DAEr(c). Please note the pronounced scattering from the micelles in c and d.

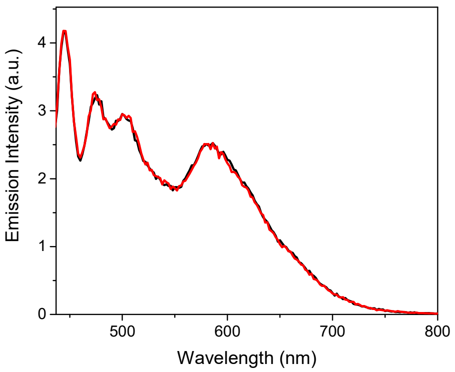

Supplementary Figure 14. Color stability. Emission spectra of the tri-component cocktail displaying emission from per, DAEg(c) and DAEr(c) before (black line) and after (red line) exposure to 423 nm excitation light for 65 minutes (corresponding to a light dose equivalent to recording 100 emission spectra at this excitation wavelength). It is obvious that the color stability is sufficiently good for the spectra not to show significant differences.

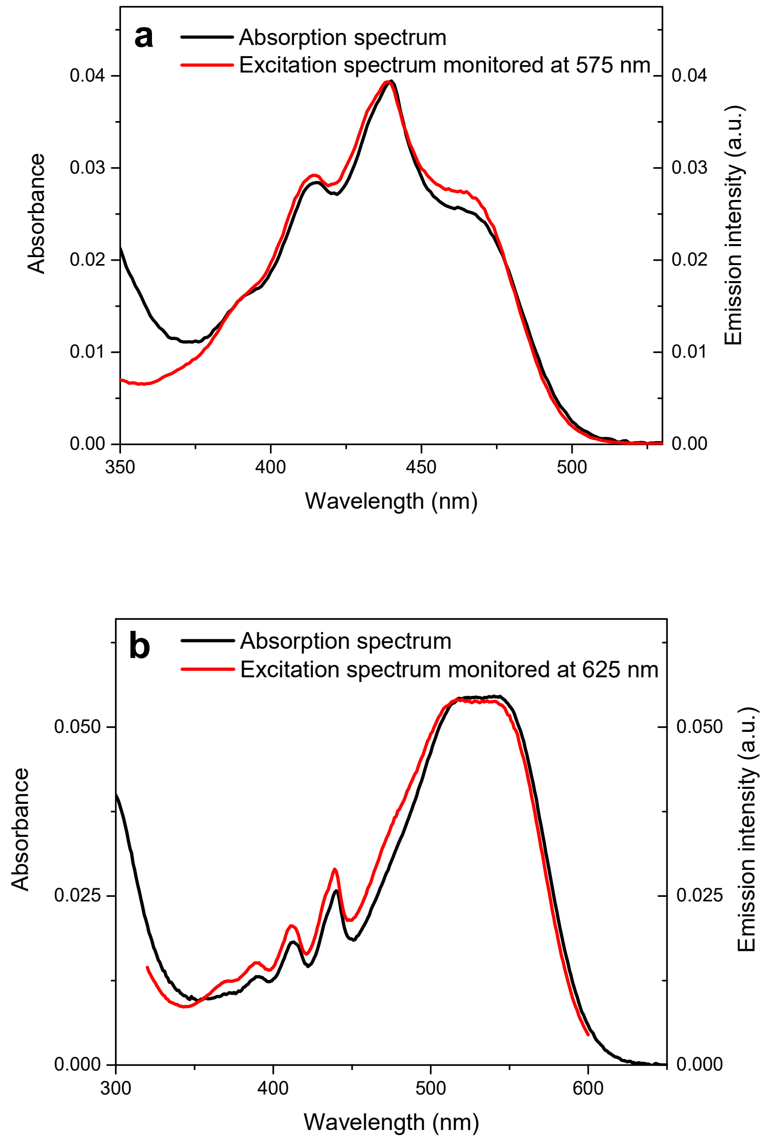

Supplementary Figure 15. FRET, absorption and excitation spectra. Absorption- (black) and excitation spectra (red) of the bi-component cocktails per-DAEg(c) (a) and per-DAEr(c) (b). The excitation spectra were recorded with the emission wavelength in regions where per does not emit. Please note that the pronounced deviations between excitation and absorption spectra observed at wavelengths shorter than ca. 400 nm is ascribed to scattering of the micelles in the absorption spectra.

Supplementary Note 5. From the data shown in Supplementary Figure 15, it is clear that FRET is the dominating quenching mechanism of per after the DAE derivatives have been isomerized to the closed form. This conclusion is based on the very similar shape of the absorption spectra and the excitation spectra of the bi-component cocktails, when the emission is monitored at a wavelength where only DAE emission is observed, known as a spectral hallmark of FRET processes with efficiencies approaching 100%.

Supplementary References

1. K. Uno, H. Niikura, M. Morimoto, Y. Ishibashi, H. Miyasaka, M. Irie, *J. Am. Chem. Soc.* **2011**, *133*, 13558-13564.

2. I. Yildiz, S. Impellizzeri, E. Deniz, B. McCaughan, J. F. Callan, F. M. Raymo, *J. Am. Chem. Soc.* **2011,** *133*, 871-879.

3. E. W. Grabner, E. Brauer, *Ber. Bunsen-Ges. Phys. Chem*. **1972**, *76*, 106-110.

4. S. Tang, B. Donaphon, M. Levitus, F. M. Raymo, *Langmuir*, **2016**, *32*, 8676-8687
